# Vagus Nerve Stimulation Promotes Epithelial Proliferation and Controls Colon Monocyte Infiltration During DSS-Induced Colitis

**DOI:** 10.3389/fmed.2021.694268

**Published:** 2021-07-07

**Authors:** Elisa Meroni, Nathalie Stakenborg, Pedro J. Gomez-Pinilla, Michelle Stakenborg, Javier Aguilera-Lizarraga, Morgane Florens, Marcello Delfini, Veronica de Simone, Gert De Hertogh, Gera Goverse, Gianluca Matteoli, Guy E. Boeckxstaens

**Affiliations:** ^1^Translational Research Center for Gastrointestinal Disorders (TARGID), Lab for Intestinal Neuro-Immune Interaction, Department of Chronic Diseases, Metabolism and Ageing, Katholieke Universiteit Leuven - University of Leuven, Leuven, Belgium; ^2^Translational Research Center for Gastrointestinal Disorders (TARGID), Lab for Mucosal Immunology, Department of Chronic Diseases, Metabolism and Ageing, Katholieke Universiteit Leuven - University of Leuven, Leuven, Belgium; ^3^Department of Pathology, Universitair Ziekenhuis Leuven, Leuven, Belgium

**Keywords:** vagus nerve stimulation, dextran sodium sulfate, inflammatory bowel diseases, macrophage - cell, epithelial barrier

## Abstract

**Background:** We previously showed increased susceptibility to dextran sulfate sodium (DSS)-induced colitis in vagotomized mice. Here, we evaluated whether vagus nerve stimulation (VNS) is able to reduce the severity of DSS colitis and aimed to unravel the mechanism involved.

**Methods:** Colitis was induced in wild type mice by 2.5% DSS administration in drinking water for 5 days. VNS (5 Hz, 1 ms, 1 mA) was applied 1 day prior to and after 4 days of DSS administration to evaluate changes in epithelial integrity and inflammatory response, respectively. Epithelial integrity was assessed using TUNEL and Ki67 staining. Monocytes, immature and mature macrophages were sorted from colonic samples and gene expression levels of pro-inflammatory cytokines were studied.

**Results:** VNS applied prior to DSS administration (i.e., prophylactic VNS) reduced disease activity index (VNS 0.8 ± 0.6 vs. sham 2.8 ± 0.7, *p* < 0.001, *n* = 5) and tended to improve histology score. Prophylactic VNS significantly increased epithelial cell proliferation and diminished apoptosis compared to sham stimulation. VNS applied at day 4 during DSS administration (i.e., therapeutic VNS) decreased the influx of monocytes, monocyte-derived macrophages and neutrophils, and significantly reduced pro-inflammatory cytokine expression (i.e., *Tnf*α and *Cxcl1*) in immature macrophages compared to sham stimulation.

**Conclusions:** A single period of VNS applied prior to DSS exposure reduced DSS-induced colitis by an improvement in epithelial integrity. On the other hand, VNS applied during the inflammatory phase of DSS colitis reduced cytokine expression in immature macrophages. Our data further underscores the potential of VNS as novel therapeutic approach for inflammatory bowel diseases.

## Introduction

More than 2 decades ago, the vagus nerve was proposed as an important regulator of the immune system. Electrical and pharmacological activation of the vagus nerve indeed improved survival in a model of sepsis by reducing TNFα production of splenic macrophages (Mφ) (1). We further extended the therapeutic potential of the so-called cholinergic anti-inflammatory pathway (CAIP) to the gut by showing that cervical and abdominal VNS dampens *muscularis* macrophage (MMφ) activation and attenuates surgery-induced intestinal inflammation (2–4). Similar to the spleen, the alpha 7 nicotinic acetylcholine receptor (α7nAChR) was identified as the target receptor of the “gastrointestinal CAIP”, suggesting that acetylcholine (ACh) released from the vagus nerve inhibits MMφ (2, 3, 5, 6). To date, electrical stimulation of the cervical vagus nerve is already used as therapeutic tool for refractory epilepsy and treatment-resistant depression (7), while its applicability in inflammatory disorders including rheumatoid arthritis (8) and inflammatory bowel diseases (IBD) (9) is currently under investigation.

IBD, comprising Crohn's disease and ulcerative colitis, consists of chronic inflammatory disorders of the gastrointestinal tract whose prevalence has dramatically increased over the past decade. IBD is the second most common chronic inflammatory disease worldwide after rheumatoid arthritis, affecting millions of people mainly in industrialized countries. Although the exact etiology is unknown, immunological, microbial, environmental, nutritional, and genetic factors are proposed to contribute to the pathogenesis and severity of IBD (10–13). To date, the clinical management has significantly improved with the introduction of biologicals, yet a significant proportion of patients remains refractory to these compounds. Based on its anti-inflammatory properties, electrical and pharmacological activation of the vagal anti-inflammatory pathway has been studied in preclinical models of IBD. Bonaz and colleagues demonstrated that 5 days of VNS successfully prevented body weight loss and improved colonic mucosal damage in a rat model of 2,4,6-trinitrobenzenesulfonic acid (TNBS) colitis (14). This effect was associated with a decrease in TNFα, IL-1β, and IL-6 levels through inhibition of the JAK2/STAT3 signaling pathway (2). In line, pharmacological activation of the CAIP was also obtained by blockade of acetylcholine esterase (AChE). In dextran sulfate sodium (DSS)-induced colitis, central activation of the vagus nerve by galantamine, an AChE inhibitor, reduced the severity of DSS-induced colitis in mice (15). Conversely, vagotomy increased the susceptibility to develop DSS colitis, impairing the ability to develop oral tolerance (16–18). However, the target population of VNS in colitis remains largely understudied.

During DSS-induced colitis, upon 3 days of DSS administration, changes in the epithelial barrier occur: increased levels of pro-inflammatory cytokines, such as TNFα, IL-1β, and IL-8 lead to a decreased barrier function inducing reorganization of tight junction proteins. These changes will ultimately trigger the immune response contributing the propagation of intestinal inflammation. Recently, Asano et al. (19) elegantly identified the kinetics of the inflammatory response triggered by DSS. In more detail, the toxic effect of DSS to the colonic epithelium induced erosions and apoptosis that eventually increased colonic epithelial permeability. In this study, it was demonstrated that a specific subset of Mφ, carrying CD169 on their surface, is strategically localized closely to the blood vessels and distant from the epithelial barrier. This unusual localization confers to the role of CD169^+^ Mφ as sentinels of the immune system in the gut, due to their ability to produce and directly release high levels of the chemoattractant CCL8, a key regulator of mucosal inflammation, into the blood stream.

Based on this study, we hypothesized that VNS reduces the severity of DSS-induced colitis not only by its anti-inflammatory properties, but also by reducing epithelial damage and/or increasing barrier resistance against DSS. Indeed, increased intestinal permeability caused by DSS allows increased entrance of antigens, resulting in an amplification of the inflammatory response in IBD (20, 21). Of interest, in a thermal injury model, post injury VNS preserves intestinal architecture by preventing epithelial barrier breakdown and reducing inflammation (22). Moreover, nicotine administration increased enteric glial cell activation, which improved intestinal epithelial barrier function in an *in vitro* model of intestinal injury (23).

Based on the above, this study was designed to evaluate the effect of VNS in DSS-induced colitis focusing on its effect on epithelial integrity in the early phase of the disease (d2), and on intestinal inflammation in a later phase of colitis (d5).

## Materials and Methods

### Experimental Animals

Ten to 14 weeks old wild-type female C57BL/6J mice were bred and housed at the KU Leuven specific pathogen-free animal facility under environmentally controlled conditions (12 h-12 h dark-light cycle; 20–22°C, 55% humidity). Mice were provided with commercially available chow (Ssniff R/M-H; ssniff Spezialdiäten GmbH, Soest, Germany) and water *ad libitum*. All animals were treated carefully in strict accordance with the ethical guidelines and all experimental procedures were approved by the Animal Care and Animal Experiments Committee of KU Leuven (P002/2014; P003/2014; Leuven, Belgium). All surgeries were performed under anesthesia and all efforts were made to minimize suffering.

### Electrical Stimulation of the Vagus Nerve

Anesthetized (2.5% isoflurane) (ISO-VET, Eurovet NV/SA, Heusden-Zolder, Belgium) mice underwent VNS as previously described (24). In more detail, a midline cervical incision was made, and the subcutaneous tissue was dissected and retracted laterally. The mandibular salivary glands were bluntly separated and retracted laterally. The right cervical vagus nerve was isolated from the carotid artery and stimulated electrically using a bipolar platinum electrode (Bilaney, Dusseldorf, Germany). Electrical stimuli consisted of square pulses (5 Hz, 1 mA, 1 ms for 5 min) (Keithley Instruments, Cleveland, Ohio) (25). Sham-operated mice were handled similarly, but the vagus nerve was not dissected from the carotid artery to avoid mechanical stimulation. Mice were kept on a heating pad to recover from surgery with *ad libitum* access to food and water. Depending on the timing of VNS application, the mice were divided into two groups: the first group of mice underwent VNS or sham-stimulation 1 day prior to DSS administration (prophylactic effect), while the second group underwent VNS or sham-stimulation after 4 days of DSS administration (therapeutic effect).

### DSS-Induced Colitis

Colitis was induced in wild-type C57BL/6J mice by administration of 2.5% DSS (TbB Consultancy AB, Uppsala, Sweden) in drinking water for 5 days followed by normal drinking water until day 14. When VNS was studied as a prophylactic treatment, VNS and sham-stimulated mice were exposed to 2.5% DSS for 2 days and sacrificed at day 2. To study the therapeutic effect of VNS during ongoing inflammation, VNS and sham-stimulated mice were exposed to 2.5% DSS for 5 days and sacrificed at day 5 by CO_2_ overdose. The colon and spleen were dissected and blood was taken *via* cardiac puncture for further investigation.

### Quantification of Disease Activity

Daily clinical assessment of DSS-treated animals included measurement of a validated clinical disease activity index (DAI), calculated using the following parameters: stool consistency (0–4), presence or absence of fecal blood (0–4) (Hemoccult, Beckman Coulter, Pasadena, USA), and weight loss (0–4) (26). After assessment of DAI at day 14, mice were sacrificed by CO_2_ overdose, and the colon was dissected for further investigation.

### Immunohistochemistry

One part of colon was immediately fixed in 4% buffered paraformaldehyde, dehydrated in 100% ethanol and embedded in paraffin. Five μm thin sections were cut. To determine the degree of colonic damage and inflammation, sections were stained with hematoxylin and eosin (H&E) and were histologically examined by a pathologist in a blinded fashion using a validated scoring system (Table 1) (27). The total colonic score was calculated as the sum of the two parameters per section (Table 1).

**Table 1:** Histological scoring using Ito et al. (27).

**Score**	**Epithelial damage (E)**	**Infiltration (I)**
0	Normal morphology	No infiltrate
1	Loss of goblet cells	Infiltrate around crypt basis
2	Loss of goblet cells in large areas	Infiltrate reaching to muscularis mucosa
3	Loss of crypts	Extensive infiltration reaching the muscularis of the mucosa with abundant edema
4	Loss of crypts in large area	Infiltration of the submucosa

*The degree of epithelial damage (E) and infiltration (I) were quantified as described, and histological score was defined as the sum of the two parameters (27)*.

In addition, other colonic sections were stained with Ki67 to detect epithelial proliferation. To this end, sections were rehydrated and blocked with 5% H_2_O_2_ and ABC Kits (Vector Laboratories, California, USA) to reduce non-specific signal from endogenous peroxidase. The slides were then incubated overnight with a rabbit polyclonal anti-Ki67 antibody (1:500, Abcam, Cambridge, UK). Cells immunoreactive to Ki67 were detected using VECTASTAIN ABC streptavidin-HRP system (Vector Laboratories, California, USA). Next, these sections were counterstained with hematoxylin, dehydrated and mounted. The number of Ki-67-positive cells was counted in 10 randomly chosen representative high-power fields. All stained sections were imaged with a microscope (BX 41 Olympus, Aartselaar, Belgium) connected to a camera (SC30 Olympus, Tokyo, Japan) and Cell^∧^F software (Olympus, Tokyo, Japan).

### Immunofluorescence

TUNEL staining was performed on colonic sections using the Click-iT TUNEL Alexa Fluor 488 Imaging Assay kit (ThermoFischer Scientific, Waltham, USA) according to the manufacturer's instructions. Samples were evaluated using confocal microscopy (Zeiss 880, ZEN software, Zeiss, Germany). The number of TUNEL-positive cells was counted in 10 randomly chosen representative high-power fields and images were acquired using a 20X objective and 40X objective.

### Isolation of Leukocytes From Blood, Spleen and Colon, and Flow Cytometry

Blood was collected by cardiac puncture. Red blood cells were lysed with homemade lysis buffer (EDTA 68.5 μM, NaHCO3 5.95 mM, NH4Cl 77.57 mM dissolved in sterile water) after which the sample was centrifuged at 2,000 *g* for 10 min. Splenocytes were obtained from homogenized spleen in phosphate-buffered saline 1X (PBS) (Lonza, Verviers, Belgium) + 1% fetal bovine serum (FBS) (Nuaillé, France). Red blood cells were lysed with homemade lysis buffer. Cell suspensions were centrifuged at 4°C, filtered with a 70 μm cell strainer and counted. Lamina propria cell suspensions were prepared from the colon lamina propria as previously described (28).

All single cell suspensions were blocked with rat anti-mouse CD16/CD32 antibodies (BD Pharmigen, Franklin Lakes, USA) (1:200) for 12 min and then incubated with the following fluorophore-conjugated antibodies at recommended dilutions for 1 h at 4°C: CD11b (M1/70; BD Biosciences), SiglecF (1RNM44N, eBioscience), Ly6G (1A8, BD Biosciences), Ly6C (AL-21, BD Biosciences), MHCII (M5/114.15.2, eBioscience), CD169 (SER4, eBioscience) to sort lamina propria monocytes (CD11b^+^SiglecF^−^Ly6G^−^Ly6C^+^MHCII^−^), immature Mφ (CD11b^+^SiglecF^−^Ly6G^−^Ly6C^+^ MHCII^+^) and mature Mφ [CD11b^+^ SiglecF^−^Ly6G^−^Ly6C^−^MHCII^+^ (CD169^−^ or CD169^+^)]. Samples were gated as indicated in Supplementary Figure 1. Dead cells were excluded using DAPI (ThermoFischer Scientific, Waltham, USA) and wash steps were performed in FACS buffer (PBS, 2% FBS and 0.78 mM EDTA). Cells were analyzed on a BD Canto HTS (BD Biosciences, Franklin Lakes, USA). Data was analyzed using FlowJo software (Tree Star Inc., Ashland, USA). FACS sorting experiments for gene expression analyses were performed on a BD Aria III (BD Biosciences, Franklin Lakes, USA) up to >95% purity. These cells were immediately sorted in RLT plus lysis buffer (QIAGEN, Hilden, Germany) containing β-mercaptoethanol and placed on dry ice.

### RNA Extraction and Gene Expression

RNA extraction was performed using RNeasy Micro Kit (Qiagen, Hilden, Germany) following the manufacturer's instructions. Total RNA was retro-transcribed into complementary cDNA using qScript cDNA SuperMix (Quanta Biosciences, Gaithersburg, USA) according to the manufacturer's instructions and gene expression was assayed by quantitative reverse transcription PCR. Quantitative real-time transcription polymerase chain reactions were performed in duplicate with the FastStart SYBR Green Master mix (Roche, Basel, Switzerland) using the LightCycler® 96 (Roche, Basel, Switzerland). The expression levels of the genes of interest were normalized to the expression levels of the reference gene *rpl32*. The mean relative gene expression was calculated using the 2^−ΔΔCT^ method. Primer sequences used are listed in Supplementary Table 1.

### Statistical Analyses

The number of animals used per group was powered based on previous experimental results and observed variability. Normality and the presence of outliers were determined *via* the Kolmogorov–Smirnov test and Grubbs' testing, respectively, prior to further statistical analysis. Two-way ANOVA test with Bonferroni correction was performed to assess the effect of VNS on body weight loss and DAI. To compare differences in gene expression, histological score and flow cytometry data between sham- and VNS-treated animals, an unpaired *t*-test or Mann-Whitney test was done depending on normality. To compare differences between naïve, sham- and VNS-stimulated mice in flow cytometry data, we performed one-way ANOVA with Sidak correction. Data are expressed as mean ± standard error of mean (SEM) or median ± interquartile range depending on normality. Probability level of *P* < 0.05 was considered statistically significant. Statistical analysis was performed with GraphPad Prism Software (GraphPad Prism, La Jolla, USA).

## Results

### VNS Prior to DSS Exposure Improves Colitis by Reducing Apoptosis and Promoting Epithelial Cell Proliferation

**Figure 1:**
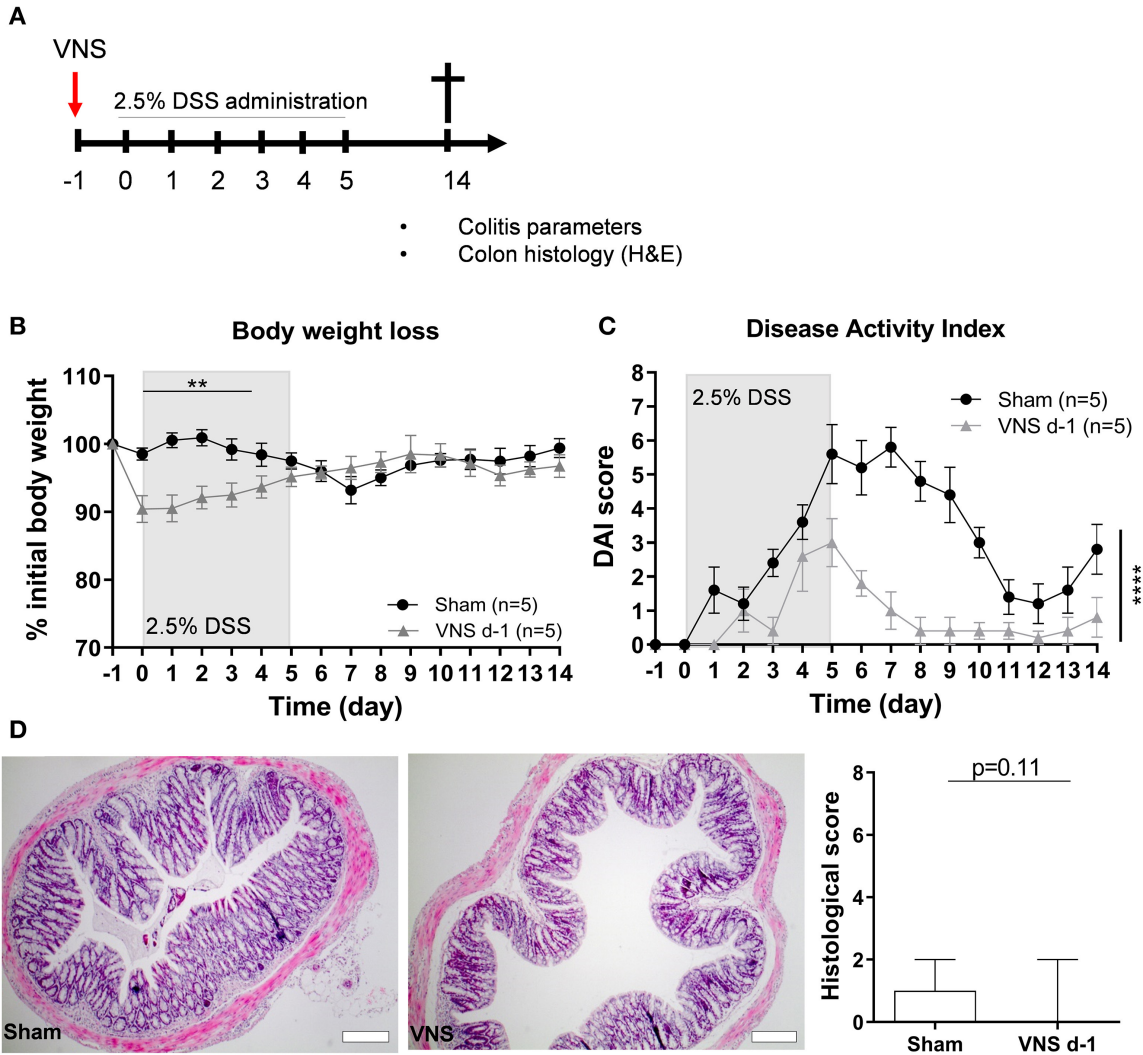
Vagus nerve stimulation prior to DSS exposure improves colitis. **(A)** Wild type mice were stimulated at day−1 and exposed to 2.5% DSS in the drinking water for 5 days, colitis severity was assessed daily. Mice were sacrificed at day 14 and tissue samples were collected for different analysis. Body weight **(B)** and disease activity index **(C)** were assessed daily. **(B,C)** Data are expressed as mean ± SEM as determined by the repeated-measures two-way ANOVA test. **p < 0.01; ****p < 0.0001; (*n* = 5 mice per group) **(D)** Representative H&E stainings of colonic sections from control sham- and VNS-stimulated mice. Scale bars are 200 μm. Bar graphs represent histological injury score of colonic samples of sham- and VNS-treated mice 14 days post colitis induction. Data are expressed as median ± interquartile range as determined by the Mann- Whitney test (*n* = 5–6 mice per group). **(A–D)** shows representative experiment with at least 1 similar replicate.

First, we evaluated if prophylactic VNS reduced the severity of DSS-induced colitis (Figure 1A). We observed that a single application of VNS 1 day prior to the induction of DSS-induced colitis (day−1) caused a significant drop in the percentage of body weight in VNS-treated animals compared to the sham-operated animals, an effect most likely caused by a combination of surgical pain and a decreased appetite (29) following the VNS procedure (data not shown). This initial body weight drop in the VNS-treated mice prevented us to observe the true beneficial effect of VNS on body weight (Figure 1B). Nevertheless, we observed that prophylactic VNS significantly improved DAI compared to sham-operated mice (Figure 1C). Moreover, VNS-treated mice tended to improve histology score compared to the sham-operated mice (Figure 1D).

**Figure 2:**
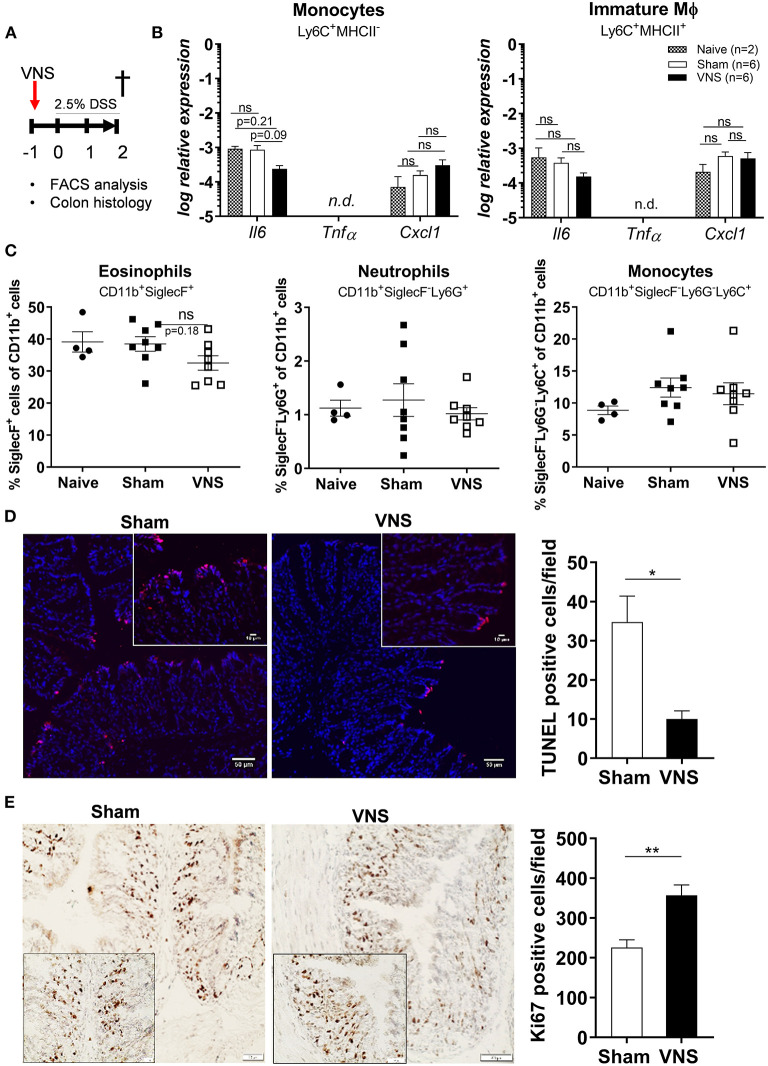
Vagus nerve stimulation tightens the epithelial barrier by reducing apoptosis and promoting proliferation. **(A)** Wild type mice were stimulated at day−1 and exposed to 2.5% DSS in the drinking water for 2 days, colitis severity was assessed daily. Mice were sacrificed at day 2 and tissue samples were collected for different analysis. **(B)** Pro-inflammatory cytokine and chemokine expression was quantified in sorted colonic monocytes and immature macrophages (Mφ) isolated from sham and VNS-treated mice at day 2. Data are expressed as mean ± SEM as determined by one-way ANOVA with multiple testing. *p < 0.05; ns, not significant; n.d., not detected; (naïve mice *n* = 2; sham-operated mice *n* = 6; VNS-treated mice *n* = 6). **(C)** Percentage of eosinophils, neutrophils, monocytes and CD169+ Mφ in lamina propria of sham and VNS-treated mice at day 2. Data are expressed as mean ± SEM as determined by the one-way ANOVA with multiple testing. **p < 0.01 (naïve mice *n* = 4; sham-operated mice *n* = 8; VNS-treated mice *n* = 8). **(D,E)** Representative images of colonic sections stained for TUNEL **(D)** and Ki67 **(E)** from sham- and VNS-stimulated mice and their relative cell count. Scale bars are 50 μm and the scale bar of the inlet is 10 μm. Bar graphs represent TUNEL-positive cells/field **(D)** and Ki67-positive cells/field **(E)** of sham- and VNS-treated mice 2 days post colitis induction. **(D,E)** Data are expressed as mean ± SEM as determined by the unpaired *t*-test (*n* = 5–6 mice per group).

As the effect of VNS only lasts 48 h (30) and intestinal inflammation is triggered from day 3 following DSS exposure (19), we hypothesized that the beneficial effect of VNS was mediated by improved resistance of the epithelium toward the cytotoxic effect of DSS. Hence, to investigate this hypothesis, mice received VNS prior to DSS and were sacrificed at day 2 (Figure 2A). To confirm that no immune activation was present at day 2, pro-inflammatory cytokine and chemokine expression was assessed in sorted monocytes and immature Mφ and the presence of different leukocyte population was determined using flow cytometry in naïve, sham-operated and VNS-treated mice. As shown in Figure 2B, no significant upregulation could be identified in both VNS-treated and sham-operated mice compared to naïve mice (monocyte *Il6* sham- vs. VNS-treated mice *p* = 0.09; *Cxcl1 p* = 0.5; immature Mφ *Il6* sham- vs. VNS-treated mice *p* = 0.3; *Cxcl1 p* = 0.8). In addition, there was no significant increase in the percentage of eosinophils, neutrophils and monocytes compared to naïve mice (Figure 2C).

Next, we evaluated epithelial damage and integrity by assessing the number of apoptotic cells (Figure 2D) and epithelial cell proliferation using TUNEL and Ki-67 stainings, respectively (Figure 2E). Of interest, VNS significantly reduced the number of apoptotic cells and increased Ki-67-positive cells compared to sham-stimulated mice.

Altogether, these data suggest that in the early phase of DSS-induced colitis, when no inflammatory response is present yet, VNS treatment has a beneficial effect on the disease course, most likely by promoting epithelial proliferation and decreasing apoptosis leading to increased resistance of the epithelium against the toxic effect of DSS.

### VNS Treatment During DSS-Induced Colitis Improves Body Weight and Disease Activity

**Figure 3:**
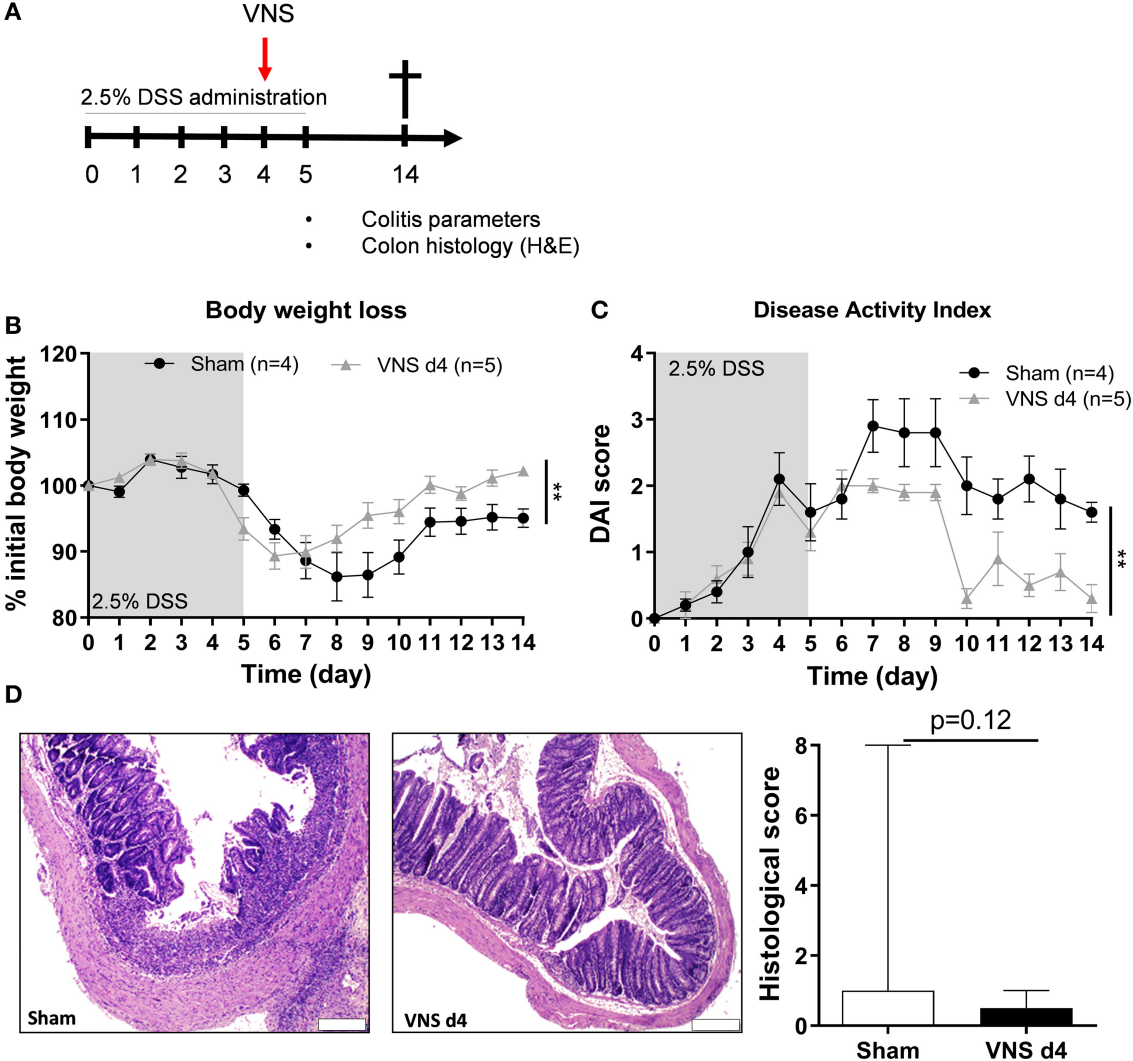
Vagus nerve stimulation during DSS colitis improves body weight and disease activity. **(A)** Wild type mice were stimulated at day 4 and exposed to 2.5% DSS in the drinking water for 5 days, colitis severity was assessed daily. Mice were sacrificed at day 14 and tissue samples were collected for different analysis. Body weight **(B)** and disease activity index **(C)** were assessed daily. Data are expressed as mean ± SEM as determined by the repeated-measures two-way ANOVA test. **p < 0.01; (sham-operated mice *n* = 4; VNS-treated mice *n* = 5). **(D)** Representative images of colonic sections stained with hematoxylin and eosin (H&E) from sham- and VNS-stimulated mice. Scale bars are 200 μm. Bar graphs represent histological injury score of colonic samples of sham- and VNS-treated mice 14 days post colitis induction. Data are expressed as median ± interquartile range as determined by the Mann- Whitney test (sham-operated mice *n* = 10; VNS-treated mice *n* = 7). **(A–D)** shows representative experiment with at least 1 similar replicate.

In the second part of this study, we aimed to evaluate the effect of VNS on the immune system during the inflammatory response. Mice received VNS or sham stimulation after 4 days of DSS exposure (Figure 3A). As shown in Figure 3B, VNS successfully prevented body weight loss compared to sham-operated mice. In line, VNS-treated mice showed a significant improvement of DAI compared to sham-operated mice (Figure 3C). Of note, VNS-treated mice only tended to have a lower histological score compared to sham-stimulated mice (Figure 3D). These findings show that VNS applied during DSS colitis is able to ameliorate the course of the disease leading to a faster recovery from colitis.

### VNS During DSS-Induced Colitis Controls Innate Immune Cell Influx Into Inflamed Colon Tissue and Dampens the Immune Response

**Figure 4:**
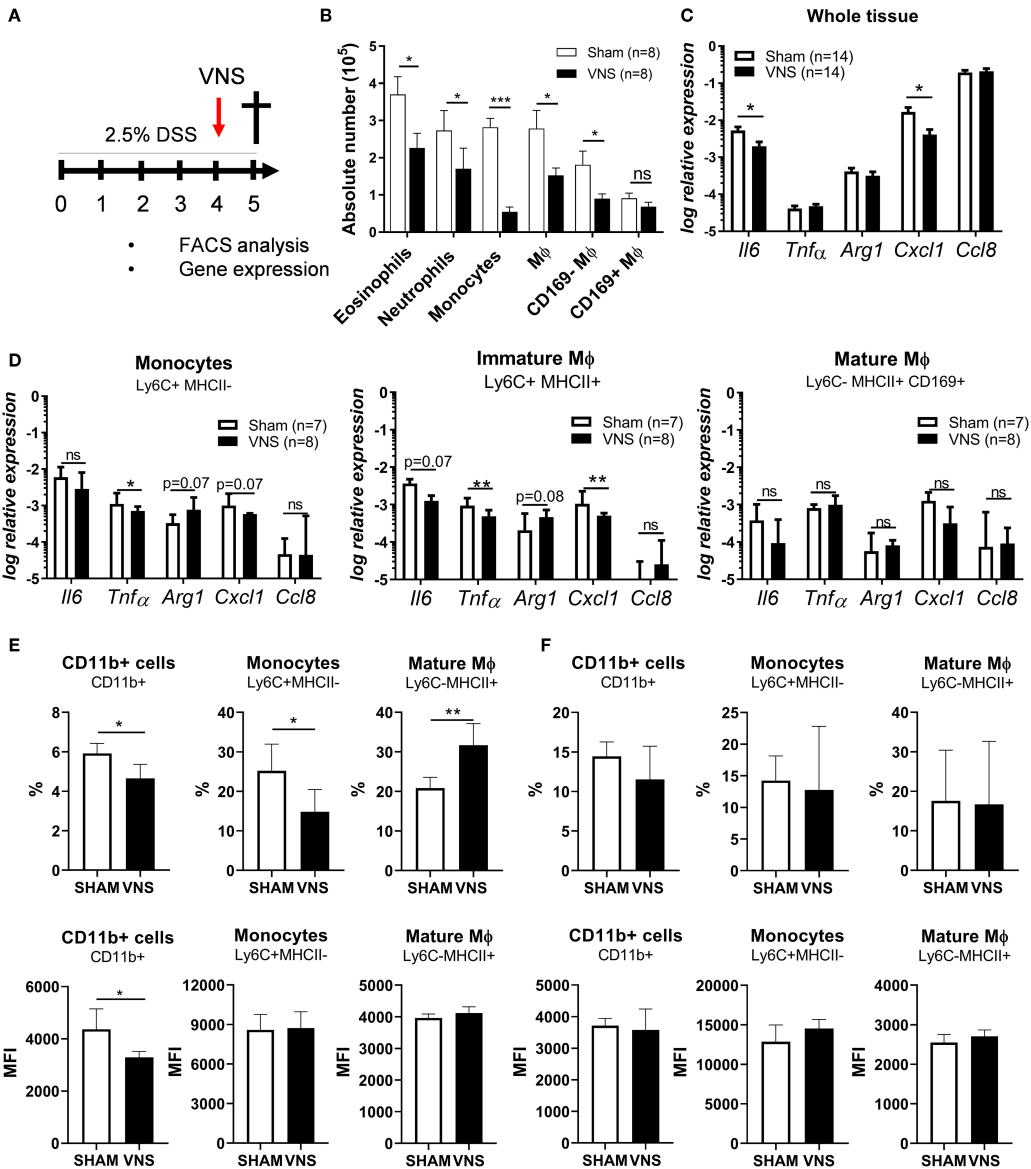
Vagus nerve stimulation affects monocyte recruitment and dampens cytokine production by immature macrophages (Mϕ) during the acute phase of colitis. **(A)** Wild type mice were stimulated at day 4 and exposed to 2.5% DSS in the drinking water for 5 days, colitis severity was assessed daily. Mice were sacrificed at day 5 and tissue samples were collected for different analysis. **(B)** Absolute numbers of eosinophils (SiglecF^+^CD11b^+^), neutrophils (Ly6G^+^CD11b^+^), monocytes (Ly6C^+^MHCII^−^) and mature macrophages (Mφ; Ly6C^−^MHCII^+^, MCHII^+^) CD169^+^ or CD169^−^ Mφ among CD11b^+^ cells from colon of sham- and VNS-treated mice 1 day post VNS. Data are expressed as mean ± SEM as determined by the unpaired *t*-test from two independent experiments. ns, not significant; *p < 0.05, **p < 0.01, ***p < 0.001; (*n* = 8 mice per group). **(C,D)** Pro-inflammatory cytokine and chemokine expression was quantified in whole tissue **(C)**; sorted colonic monocytes, immature Mφ and mature CD169^+^ Mφ **(D)** isolated from sham and VNS-treated mice at day 5. Data are expressed as median ± interquartile range as determined by the Mann-Whitney test from two independent experiments. *p < 0.05, **p < 0.01; ns, not significant; (*n* = 7–14 mice per group). Data representative of two independent experiments. **(E,F)** The percentage and MFI of splenic **(E)** and blood **(F)** CD11b+ cells, monocytes (Ly6C^+^MHCII^−^) and mature Mφ (Mφ; Ly6C^−^MHCII^+^, MCHII^+^) from sham and VNS-treated mice at day 5. Data are expressed as mean ± SEM as determined by unpaired *t*-test. *p < 0.05, **p < 0.01 (sham-operated mice *n* = 5; VNS-treated mice *n* = 5).

Next, we aimed to study the effect of VNS on the immune response in more detail and to characterize the target cell(s) population affected by VNS. Therefore, neutrophils, monocytes, eosinophil, immature Mφ and mature CD169^+^ and CD169^−^ Mφ from the colonic lamina propria of day 4 VNS- and sham-stimulated mice were studied and characterized by flow cytometry (Supplementary Figure 1 and Figures 4A,B). As shown in Figure 4B, VNS-treated mice showed a decreased number of eosinophils, neutrophils, monocytes, immature Mφ and mature CD169^−^ Mφ in the lamina propria compared to sham-operated mice. CD169^+^ Mφ were however not significantly decreased. Similar to whole colon tissue, immature Mφ of VNS-treated mice showed decreased gene expression of *Tnf*α and *Cxcl1* compared to sham-operated mice (Figures 4C,D), while VNS did not affect the pro- and anti-inflammatory cytokine expression in the CD169^+^ Mφ population. Previous studies also found that electrical VNS dampened leukocyte trafficking (31). To this end, we examined if VNS altered the expression of surface markers associated with migration such as CD11b in the spleen and blood. Interestingly, VNS-treated mice had a reduced percentage and mean fluorescent intensity (MFI) of CD11b+ cells and monocytes in the spleen compared to sham-operated animals (Figure 4E), while the percentage and MFI of CD11b+ cells and monocytes remained unchanged in blood (Figure 4F). These observations suggest that a reduction in surface CD11b levels by VNS might contribute to the observed reduction in inflammatory cells in the intestine. Altogether, these data demonstrate that VNS modulates the inflammatory response during colitis by reducing the influx of inflammatory cells, such as eosinophils, neutrophils, incoming monocytes, and immature Mφ.

## Discussion

In the past decades, preclinical studies have demonstrated the beneficial effect of the CAIP in several inflammatory disorders. In the present study, we investigated the prophylactic and therapeutic effect of VNS in a model of DSS-induced colitis. Our results showed that prophylactic VNS was associated with lowered DAI compared to sham-operated animals. These improved clinical parameters are most probably due to the maintenance of the intestinal barrier by the induction of epithelial cell proliferation and decreased cell apoptosis. Of note, prophylactic VNS leads to significant body weight loss compared to the sham-operated animals, an effect most likely due to decreased appetite following the VNS surgical procedure rendering it difficult to appreciate the true beneficial effect of prophylactic VNS in the early disease course of DSS-induced colitis. Nevertheless, we found that therapeutic VNS also decreased DAI and tended to reduce histological score compared to sham-operated animals, which is due to a decreased cytokine expression in incoming monocytes and differentiating Mφ, an effect associated with reduced influx of immune cells into the gut. These observations highlight the fact that VNS should be further investigated as a possible prophylaxis of IBD flares as well as therapeutic treatment during active IBD.

Based on a previous study by Asano et al. (19), we hypothesized that VNS might act through two distinct mechanisms. We proposed that during the initial phase of the disease (i.e., first 48 h), VNS improved the maintenance of intestinal epithelial barrier integrity, which is vital to limit bacterial translocation across the membrane and to prevent a subsequent systemic inflammatory response. Altered intestinal barrier integrity is not only associated with major trauma and septic states, but it is also integral in many intestinal diseases including IBD, stress-induced conditions such as irritable bowel syndrome, food allergy, and other intestinal infections (32). During these pathological conditions, mechanisms such as tight junction rearrangements, decreased mitochondrial function, increased apoptosis, and diminished proliferation of the epithelium can all contribute to barrier disruption (33). In the present study, we showed that prophylactic treatment with VNS significantly increased epithelial cell proliferation and diminished apoptosis compared to sham stimulation. These findings are in line with several previous studies evaluating the effect of VNS on gut barrier function. For instance, in a model of skin burn injury, VNS prevented intestinal barrier injury by decreasing intestinal levels of TNF-α, a phenomenon associated with modulation of the intestinal tight junction proteins, MLCK and MLC, and known to decrease intestinal epithelial permeability (22, 34). Similarly, VNS protects tight junction expression and barrier function in endotoxemic mice and gut ischemia by suppressing NF-kB translocation and downregulating MLCK (33, 35). In our study, no inflammation could be demonstrated after 2 days of DSS treatment, excluding the possibility that a reduction in the activation of the NF-kB pathway and the release of TNF-α or IL-8 can explain the improvement in barrier function. Of interest, however, VNS was previously reported to reduce the amount of intracellular mucus in the tracheal goblet cells of guinea pigs, suggesting increased mucus secretion (36). To what extent increased mucus production contributes to the increased protection against DSS observed in our study remains to be investigated. Of note, however, we showed that VNS applied prior to DSS administration reduces apoptosis and promotes proliferation of intestinal epithelial cells, leading to a milder development of colitis. We speculate that these mechanisms contribute to a better protection against the noxious effect of DSS and may be of interest to prevent disease recurrence in IBD patients, although more experiments are required to further explore this hypothesis.

To investigate if VNS is also effective in reducing active disease, we evaluated the effect of VNS during ongoing DSS colitis. Of interest, we showed that VNS applied on day 4 of DSS administration improved disease outcome, associated with reduced body weight loss and a tendency to restore the normal colonic architecture of the intestine. Importantly, through flow cytometric analysis we observed a significant decrease in the influx of innate immune cells, in particular monocytes, after VNS compared to sham-stimulated mice. As CCL8 produced by CD169^+^ Mφ has been proposed to be detrimental in attracting monocytes and pro-inflammatory Mφ, we focused on this cell population as potential target of VNS. However, no effect of VNS could be detected on CCL8 expression by CD169^+^ Mφ arguing against the hypothesis that a reduction of CCL8 production by CD169^+^ Mφ with subsequent prevention of influx of monocytes and pro-inflammatory Mφ explains the anti-inflammatory effect of VNS in DSS-induced colitis. Instead, we observed decreased expression of the pro-inflammatory cytokines *Tnf*α and *Cxcl1* in Ly6C^+^ MHCII^+^ immature Mφ, indicating that these immune cells represent the potential target cells of VNS. The degree of reduction in gene expression was however rather limited suggesting that additional mechanisms might be responsible for the significant reduction in incoming immune cells. Of interest, VNS was also shown to reduce leukocyte trafficking *via* down-regulation of CD11b, a β2-integrin involved in cell adhesion and leukocyte chemotaxis, a mechanism that takes place in the spleen (31). Alternatively, previous studies have proposed that the anti-inflammatory effect of VNS in experimental DNBS colitis is mediated by altered dendritic—T cell interaction in the spleen (37). This mechanism is however unlikely to explain our findings as DSS colitis is a T cell-independent model of colitis. So clearly, more experiments are required to further explore the underlying mechanisms of the anti-inflammatory effect of VNS in DSS colitis.

In summary, our results show that VNS can improve the maintenance of the intestinal epithelial barrier by promoting epithelial cell proliferation and decreasing cell apoptosis. Secondly, VNS decreases cytokine expression in incoming monocytes and Ly6C^+^ MHCII^+^ Mφ, an effect associated with a reduction in influx of immune cells. Our preclinical findings underscore that VNS might be an interesting therapeutic approach to treat inflammatory disorders such as IBD. With this study, we shed new light on the mechanism of action of VNS and its cellular target, however, the exact molecular mechanisms still need to be further investigated.

## Data Availability Statement

The raw data supporting the conclusions of this article will be made available by the authors, without undue reservation.

## Ethics Statement

The animal study was reviewed and approved by Animal Care and Animal Experiments Committee of KU Leuven.

## Author Contributions

EM, NS, GM, and GB planned and designed experiments. MS, JA-L, MF, MD, VS, GD, GG, and GM performed or supervised the experiments. EM, NS, and GB reviewed data and wrote the manuscript. All authors corrected and approved the final version of the manuscript.

## Conflict of Interest

The authors declare that the research was conducted in the absence of any commercial or financial relationships that could be construed as a potential conflict of interest.
