# Supplementary material for: Vagus Nerve Stimulation Promotes Epithelial Proliferation and Controls Colon Monocyte Infiltration During DSS-Induced Colitis
Source: Front Med (Lausanne). 2021 Jul 7;8:694268. doi: 10.3389/fmed.2021.694268 (PMC8292675; doi:10.3389/fmed.2021.694268)
Supplement: Supplementary file 1 [file Data_Sheet_1.docx]

**Title: Vagus nerve stimulation promotes epithelial proliferation and controls colon monocyte infiltration during DSS-induced colitis**

**Authors: Elisa Meroni**^1*^; **Nathalie Stakenborg^1*^**; Pedro J. Gomez-Pinilla^1^; Michelle Stakenborg^2^; Javier Aguilera- Lizagarra^1^; Morgane Florens^1^; Marcello Delfini^1^; Veronica de Simone^2^; Gert De Hertogh^3^, Gera Goverse^2^; Gianluca Matteoli^2^; Guy E. Boeckxstaens^1^

^1^ KU Leuven - University of Leuven, Department of Chronic Diseases, Metabolism and Ageing, Translational Research Center for Gastrointestinal Disorders (TARGID), Lab for Intestinal Neuro-immune interaction, Leuven, Belgium.

^2^ KU Leuven - University of Leuven, Department of Chronic Diseases, Metabolism and Ageing, Translational Research Center for Gastrointestinal Disorders (TARGID), Lab for Mucosal Immunology, Leuven, Belgium.

^3^ Department of Pathology, UZ Leuven, Belgium

*These authors equally contributed to this work.

**Corresponding author:**

Prof. Guy E. Boeckxstaens

Herestraat 49, O&N1 bus 701

3000 Leuven, Belgium

Tel.: +32 16 377566

email: Guy.Boeckxstaens@kuleuven.be

**Conflict of interest:** The authors declare to have no conflict of interest.

**Author contribution:** EM, NS, GM and GEB planned and designed experiments MS, JAL, MF, MS, VS, GDH, GG and GM performed or supervised the experiments. EM, NS and GEB reviewed data and wrote the manuscript. All other authors corrected and approved the final version of the manuscript.

**Funding:** This work was supported by the European Research Council (ERC) Advanced Grant (ERC-340101-Cholstim) to GEB. GEB is also supported by Flanders Fund for Innovation by Science and Technology (IWT-TBM; 110699), and Research Foundation—Flanders (FWO): Odysseus programme (G.0905.07) and two FWO grants (G.0566.12N and G.0890.18N). NS (12V3619N), PJG-P, JAL (12X9820N) and GG are supported by a postdoctoral research fellowship of FWO. MS is supported by a PhD fellowship of FWO. GM is supported by an FWO grant (G.0D83.17N) and by KU Leuven grants (ZKD2906-C14/17/097 and ZKC9531-C12/15/016).

**Acknowledgements:** The authors would also like to thank I. Appeltans for her excellent technical assistance. Tissue imaging was performed at the Cell Imaging Core (KU Leuven, Belgium). Cell sorting and flow cytometry was done at the FACS core facility (KU Leuven, Belgium).

**Supplementary information**

**Supplementary Table1. Primer sequences used for qRT-PCR**

| ***Gene*** | ***Sense*** | ***Antisense*** |
| --- | --- | --- |
| ***Rpl32*** | 5’-AAGCGAAACTGGCGGAAAC-3’ | 5’-TAACCGATGTTGGGCATCAG-3’ |
| ***Il6*** | 5’-CCATAGCTACCTGGAGTACATG-3’ | 5’-TGGAAATTGGGGTAGGAAGGAC-3’ |
| ***Tnfα*** | 5’-TCTTCTCATTCCTGCTTGTGG-3’ | 5’-CACTTGGTGGTTTGCTACGA-3’ |
| ***Arg1*** | 5’-TCACCTGAGCTTTGATGTCG-3’ | 5’-TTATGGTTACCCTCCCGTTG-3’ |
| ***Ccl8*** | 5’-TCTACGCAGTGCTTCTTTGC-3’ | 5’-ATACCCTGCTTGGTCTGGAA-3’ |
| ***Cxcl1*** | 5’- GCTGGGATTCACCTCAAGAA-3’ | 5’-TCTCCGTTACTTGGGGACAC-3’ |

**Supplementary Figure 1. Gating strategy of the immune profile characterization of the inflammatory response during prophylactic VNS treatment.** (A) Representative expression of DAPI and CD11b (left column), Ly6G and CD11b (mid-left column), SiglecF and CD11b (mid column), Ly6C and MHCII (mid-right column) and MHCII and CD169 (right column) from colon of Sham- and VNS-treated mice 3 days post VNS.
